# Development and Characterization of Physical Modified Pearl Millet Starch-Based Films

**DOI:** 10.3390/foods10071609

**Published:** 2021-07-12

**Authors:** Sneh Punia Bangar, Manju Nehra, Anil Kumar Siroha, Michal Petrů, Rushdan Ahmad Ilyas, Urmila Devi, Priyanka Devi

**Affiliations:** 1 Department Food, Nutrition and Packaging Sciences, Clemson University, Clemson, SC 29631, USA; 2 Department Food Science and Technology, Chaudhary Devi Lal University, Sirsa 125055, Haryana, India; manju.vnehra@gmail.com (M.N.); 9728581773urmi@gmail.com (U.D.); ahlawatpriyanka755@gmail.com (P.D.); 3 Institute for Nanomaterials, Advanced Technologies and Innovation (CXI), Technical University of Liberec, Studentská 2, 461 17 Liberec, Czech Republic; michal.petru@tul.cz; 4 Faculty of Engineering, School of Chemical and Energy Engineering, Universiti Teknologi Malaysia, UTM, Johor Bahru 81310, Johor, Malaysia; ahmadilyas@utm.my; 5 Centre for Advanced Composite Materials (CACM), Universiti Teknologi Malaysia, UTM, Johor Bahru 81310, Johor, Malaysia

**Keywords:** pearl millet starch, physical modification, physicochemical, morphology, particle size

## Abstract

Pearl millet is an underutilized and drought-resistant crop that is mainly used for animal feed and fodder. Starch (70%) is the main constituent of the pearl millet grain; this starch may be a good substitute for major sources of starch such as corn, rice, potatoes, etc. Starch was isolated from pearl millet grains and modified with different physical treatments (heat-moisture (HMT), microwave (MT), and sonication treatment (ST)). The amylose content and swelling capacity of the starches decreased after HMT and MT, while the reverse was observed for ST. Transition temperatures (onset (To), peak of gelatinization (Tp), and conclusion (Tc)) of the starches ranged from 62.92–76.16 °C, 67.95–81.05 °C, and 73.78–84.50 °C, respectively. After modification (HMT, MT, and ST), an increase in the transition temperatures was observed. Peak-viscosity of the native starch was observed to be 995 mPa.s., which was higher than the starch modified with HMT and MT. Rheological characteristics (storage modulus (G′) and loss modulus (G′′)) of the native and modified starches differed from 1039 to 1730 Pa and 83 to 94 Pa; the largest value was found for starch treated with ST and HMT. SEM showed cracks and holes on granule surfaces after HMT as well as MT starch granules. Films were prepared using both native and modified starches. The modification of the starches with different treatments had a significant impact on the moisture, transmittance, and solubility of films. The findings of this study will provide a better understanding of the functional properties of pearl millet starch for its possible utilization in film formation.

## 1. Introduction

Pearl millet is a drought-resistant crop that belongs to the grass family, the *Poaceae,* and is a native African millet that is cultivated in the arid and semiarid tropics of Africa and Asia. The worldwide production of millet is 31,019,370 tonnes, and India is top producer of millet (11,640,000 tonnes) followed by Niger (3,856,344) and Nigeria (2,240,744 tonnes) [1]. Because of its dearth of industrial applications, it is an economical source for the isolation of starch. Modification and isolation of the starch from pearl millet can provide a new direction for food industries.

Native starch had some drawbacks such as the lesser stability of the starch paste when given high shear, acidic, or freezing conditions, which affects the quality of food products [2,3]. As such, by applying different methods of modification, the application of starch in various industries can be increased. By improving the qualities of the starch without using any chemicals to induce starch modification, more natural food components can be produced [4]. Due to the safe and natural materials used for the physical modification of starches, the amount used in food application is not limited by legislation. Ambigaipalan et al. [5] stated that heat moisture treatment (HMT) rearranges the starch molecules and hence improves their functional properties such as high gelatinization temperature, limited swelling, and stable paste viscosity. During the modification of starch with HMT, the starch is treated with both moisture (10–35%) and temperature (90–120 °C) for a specific time period [6].

Microwave treatment (MT) is one of the starch modification techniques which can be used to change the functional characteristics of starches [7]. Microwaves are non-ionizing electromagnetic radiation that alter the characteristics of a material through dielectric heating and electromagnetic polarization effects [8]. It has been reported that MT can induce crystal collapse and accelerate the rearrangement of crystalline regions, which can alter the water absorption capacity, paste viscosity, relative crystallinity, and digestion behavior of starch [9]. Another innovative approach for the modification of starch is ultrasound (>15–20 kHz). It is generally used for the gelatinization of native starch granules dispersed in a liquid, usually water. Ultra-sonication treatment changes the properties of starches, and these changes are influenced by many components, such as starch source, ultrasound power and frequency, time, and temperature [10].

The preparation of films and coatings based on polysaccharides, such as starch, has shown great growth in recent years due to the interesting mechanical and barrier properties observed in films prepared from these materials [11]. Dai et al. [12] explained that starch is a natural source of polysaccharides, which have many benefits such as good availability, renewability, and biodegradability; they especially have good film-forming characteristics. As such, starch is the main resource used in the preparation of biodegradable films. Limited information is available on the physical modifications (HMT, MT, and sonication treatment (ST)) of pearl millet starch and its applications; therefore, this study was conducted to evaluate the physicochemical, rheological, and morphological properties of physical modified starches and their applications in film preparation.

## 2. Materials and Methods

### 2.1. Materials

Pearl millet cultivar (HC-20) was collected from Chaudhary Charan Singh Haryana Agricultural University, Hisar (HR), India. The chemicals used were of analytical grade.

### 2.2. Methods

#### 2.2.1. Starch Extraction

Sandhu and Singh’s [13] method opted for the isolation of starch from pearl millet. Pearl millet grains were soaked (0.1% Na_2_S_2_O_5_, 18–20 h) and ground in a grinder (Sujata Powermatic Plus, New Delhi, India). To remove the protein and fibrous matter, the slurry was passed through nylon cloth (100 mesh) and centrifuged (605× *g*, 10 min). To dry the starch, a universal oven (NSW, New Delhi, India) was used (45 °C, 12 h).

#### 2.2.2. Starch Modification

##### Heat Moisture Treatment (HMT)

Pearl millet starch was treated with HMT using the method of Collado [14]. The moisture content of the starch was adjusted to 25% with distilled water and mixed properly. The starch was placed in a baking pan and covered with aluminum foil and kept overnight at 4–6 °C. Starch was kept in the oven for 3 h at 110 °C and shaken irregularly for the equal circulation of heat. After the desired time period (3 h), the sample was dried in the oven (50 °C, 12 h) after cooling at room temperature.

##### Microwave Treatment

For MT, starch moisture content was adjusted to 25% with distilled water and mixed properly. Vacuum bags were used to hold the starch to be used for MT, and the bags were vacuumed, pressed to ensure uniform thickness (3.5 mm), and kept for 24 h at 4–6 °C before microwave treatment [15].

##### Sonication Treatment

ST was applied to starch by following the method of Majeed et al. [16]. A starch suspension was prepared (1:10 ratio) using distilled water and subjected to ST in a low-power sonicator with a frequency of 33 kHz (JSGW, New Delhi, India) for 45 min followed by centrifugation to remove the water and dried at 50 °C (12 h).

#### 2.2.3. Physicochemical Characteristics

The amylose content of the starches was evaluated using the method of Williams et al. [17]. The swelling and solubility of the starches were evaluated by opting for the method of Leach et al. [18]. The method of Perera and Hoover [19] was used to calculate the transmittance of starches. A starch solution (1%) was prepared by heating the suspension at 90 °C for 1 h with constant stirring. The transmittance of the sample was measured at 640 nm using water as a blank at different times (0, 24 h, 48, 72 h, 96 h, and 130 h) after being stored at 4 °C in a refrigerator.

#### 2.2.4. Thermal Characteristics

Thermal characteristics of the starches were analysed using a DSC (Mettler Toledo, Greifensee CH-8606, Switzerland). For measurement, a starch to water ratio 1:3 (*w*/*w*) was added to an aluminium pan, and an empty pan was used as reference. Pans were hermetically sealed and heated from 40 to 150 °C at the rate of 5 °C/min. Thermal transitions of the starch samples were defined as To (onset), Tp (peak of gelatinization), and Tc (conclusion), and ∆Hgel referred to the enthalpy of gelatinization. Enthalpies were calculated on the basis of dry starch. These were calculated automatically. The gelatinization temperature range (R) and peak height index (PHI) were calculated as 2(Tp-To) and ∆H/(Tp-To), respectively.

#### 2.2.5. Pasting Characteristics

Pasting characteristics of samples were measured by using an in-build starch cell in Rheometer (MCR-52, Anton Paar, Graz-8054, Austria). The starch suspension (8%) was equilibrated at 50 °C for 1 min. and heated at 6 °C/min to 95 °C. After holding at 95 °C (2.7 min), the suspension was cooled to 50 °C at 6 °C/min and held at 50 °C for 2 min. Peak viscosity (PV), breakdown (BV), trough (TV), setback (SV), final viscosity (FV), and pasting temperature (PT) were obtained from the pasting graph.

#### 2.2.6. Rheological Characteristics

Rheological characteristics of samples were calculated with rheometer (MCR-52). A parallel plate system was used (4 cm diameter) and a gap size of 1000 µm was maintained throughout the study. Starch paste (10%) was prepared by heating the starch suspension at 85 °C in a water bath for three minutes. Before loading the sample on the rheometer, the sample was cooled to room temperature. A frequency sweep test was performed from 0.1–100 rad/s at 25 °C. The storage modulus (G′), loss modulus (G′′), and damping factor (tanδ) were evaluated using the graph.

#### 2.2.7. X-ray Diffraction (XRD)

XRD pattern of starches were analyzed using the diffractometer (Rigaku Miniflex, Tokyo, Japan). Diffractograms were evaluated at 25 °C over a 2θ range of 4–40 with a step size of 0.02 and a sampling interval of 10 s.

#### 2.2.8. Morphological Characteristics

Morphological characteristics were evaluated using SEM (Model EVOLS10 ZEISS, Oberkochen, Germany). A starch suspension (1%) was prepared using ethanol and the sample was loaded onto an aluminium stub and was coated with gold–palladium (60:40). During analysis, an acceleration potential of 10 kV and a magnification power 1500× were used.

#### 2.2.9. Particle Size Distribution (PSD)

PSD of the starch was analyzed using a laser diffraction particle size analyzer (Malvern Instruments Ltd., Zeta sizer version, 7.11, Malvern, UK). Sonication (3 min) was applied to the starch sample prior to estimation. The refractive indexes were 1.33 and 1.53 for distilled water and starch during analysis.

#### 2.2.10. Film Formation

Starch films were prepared by following the method of da Rosa Zavareze et al. [20] with some modifications. A starch slurry (4%) was heated at 90 °C for 10 min and glycerol 1% (*w*/*w*) was added after cooling the starch slurry to room temperature and stirred at 150 rpm for 20 min. After passing the starch slurry through a muslin cloth, solutions were cast on Teflon coated baking trays and dried in the oven (NSW, New Delhi, India) at 50 °C for 16 h. After cooling, the films were peeled off and stored at 25 °C/53% relative humidity for 48 h for further measurements.

#### 2.2.11. Characteristics of Film

The method of Galus et al. [21] was opted for to determine the moisture content of starch film. Vernier Calliper was used to determine the thickness of starch films [22]. The water solubility of the film was calculated using the method described by Gontard et al. [23]. Film opacity was measured using a UV-visible spectrophotometer (Systronics, Ahmadabad, India). Opacity of the film was calculated at 600 nm using an empty cuvette as a blank and was calculated using method of Han and Floros [24].

#### 2.2.12. Statistical Analysis

Triplicate observations were submitted to one-way analysis of variance (ANOVA) using Minitab Statistical Software version 15 (Minitab Inc., State College, PA, USA).

## 3. Results

### 3.1. Physicochemical Characteristics

Amylose content (AC) plays a key role in the characteristics and applications of starch. Different methods are applied for the calculation of AC, but the iodine binding spectrophotometry method is a basic method for the determination of AC [25]. In this investigation, AC was calculated using the iodine binding spectrophotometry method, and the AC of the native starch was found to be 18.2% (Table 1). AC was reduced after HMT and MT, while the reverse was observed for ST. Similar results for the reduced AC of HMT pearl millet starches were reported by Sandhu et al. [26]. Chan et al. [27] observed an increase in AC after ST. This increase may be due to the partial de-polymerization and the molecular scission of chains caused by the modification, which results in the increase of small chains. Swelling power (SP) and solubility give an indication of the degree of interaction between starch chains within the amorphous and crystalline domains [28]. The rheological characteristics of starch (thickening behaviour) are significantly influenced by the magnitude of SP and the solubility of starch [29]. The SP of native starch was observed to be 15.86 g/g, which was higher than HMT (12.64 g/g) and MT starch (12.63 g/g) and lower than ST starch (17.53 g/g), respectively (Table 1). Similar results for HMT starches were evaluated by Sandhu et al. [26]. Carmona-García et al. [30] explained the increase in SP after ST. When ST time increases, it results in the disorganization of the starch constituents due to the leaching of AC, which enhances the water absorption capacity of the starch, thereby increasing the SP. SP reduced after HMT due to increased crystallinity, amylose–lipid interactions, and/or interactions involving amylose–amylose and/or amylose–amylopectin chains [6,31]. A decrease in SP after MT may be due to the reorganization of crystalline areas within the starch granules, which might become more randomly distributed within the starch granule [32].

**Figure 1:**
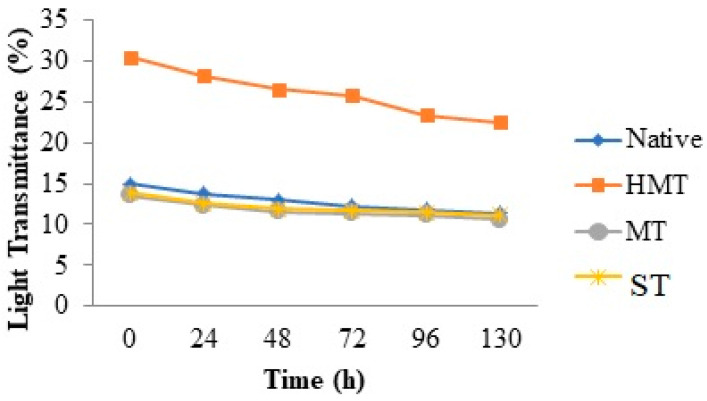
Light transmittance of native and modified starches.

The light transmittance of starches ranged between 13.53 to 30.52%. The largest value was evaluated for HMT starch. Light transmittance was measured for the storage period of 130 h at 4 °C (Figure 1). With an increase in the time, a decrease in light transmittance was observed. Paste clarity describes the light transmittance ability of a starch suspension when light passes through it, and paste clarity depends on the swelling behaviour of starch granules [13].

### 3.2. Thermal Characteristics

The thermal analysis data for the native and modified starches are tabulated in Table 2. When starch is heated in excess water, an irreversible phase transition process known as gelatinization occurs. The cooking properties of starches are calculated by gelatinization of the starch, which has an important role in food processing, and starches that had low gelatinization temperatures showed better cooking characteristics [33]. Transition temperatures (onset (To), peak of gelatinization (Tp), and conclusion (Tc)) of the starches varied from 62.92–76.16 °C, 67.95–81.05 °C, and 73.78–84.50 °C, respectively. After modification (HMT, MT & ST), an increase in the transition temperature was observed. The enthalpy of gelatinization (∆Hgel) of the starches varied from 7.9 to 10.8 J/g. The largest and the smallest values were observed for native and HMT starch. Shiotsubo and Takahashi [34] explained that ΔH showed the loss of the double-helix and molecular order in the crystallite region. The lesser values showed less organization of crystalline region, and the stability of the granule structure is also shown to decrease. Sharma et al. [35] reported the thermal properties of HMT millet starch at various moisture contents (20–30%) and observed an increase in gelatinization temperatures as the amount of moisture was increased. After MT, significant increases in gelatinization temperatures were evaluated, and this increase was positively related with the moisture content of MT [15]. Falsafi et al. [36] reported the impact of different power and sonication times on the thermal characteristics of oat starch and the observed increase in the gelatinization temperature.

### 3.3. Pasting Characteristics

**Figure 2:**
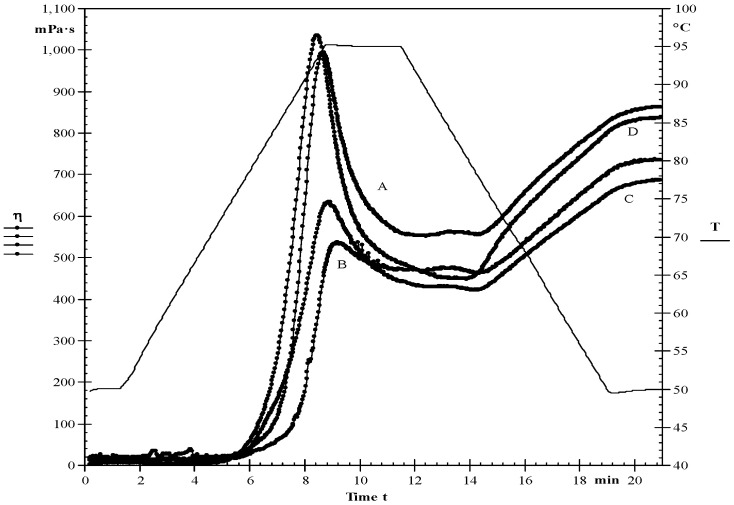
Pasting properties of native and modified starches. (**A**) Native starch (**B**) HMT (**C**) Microwave (**D**) Sonication.

Significant (*p* < 0.05) modification impacts were found on the pasting characteristics of starches (Figure 2 & Table 3). The PV of native starch was observed to be 995 mPa.s, the PV of HMT starch (536 mPa·s) and MT starch (630 mPa·s) were observed to be lower than ST starch (1034 mPa·s). Similar observations for PV were reported by Li et al. [15] and Sandhu et al. [26]. Due to the destruction of starch molecules by physical modification methods, a decrease in viscosity occurs. BV showed granule stability and starch paste consistency during shearing at higher temperatures [37]. The BV of native starch was observed to be 443 mPa·s, which was higher compared to the HMT (74 mPa·s) and MT (209 mPa·s) starches. PT provides information about the cooking behaviour of starches; starch that had less PT required a lower cooking temperature and had lower resistance to swelling and rupture. The reduced SV observed for HMT and MT starches when compared to native starch indicates a reduced tendency for retrogradation. The PT of native starch was found to be 72.8 °C, which was less than the HMT (75.2 °C), MT (77.0 °C), and ST starch (73.4 °C), respectively.

### 3.4. Rheological Characteristics

**Figure 3:**
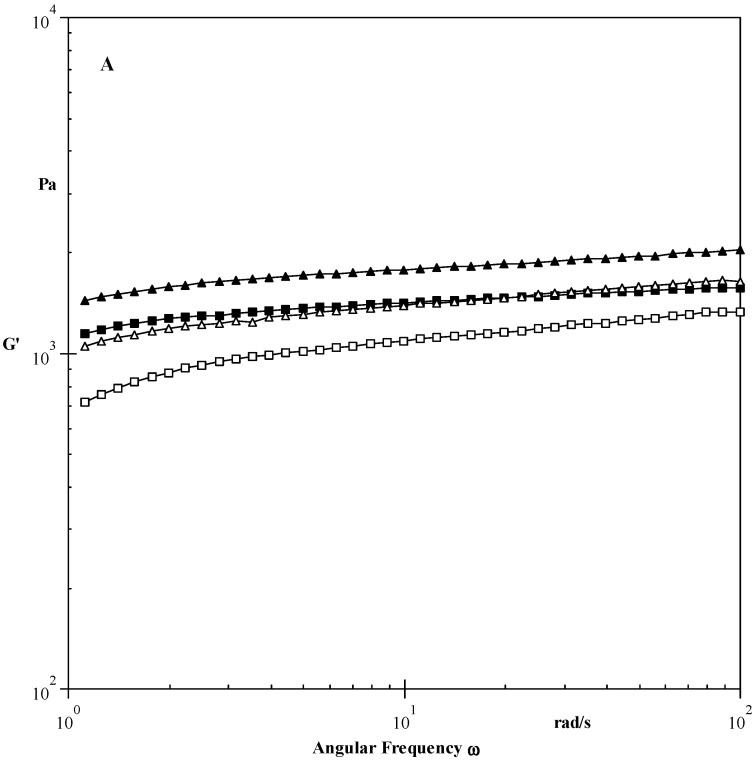

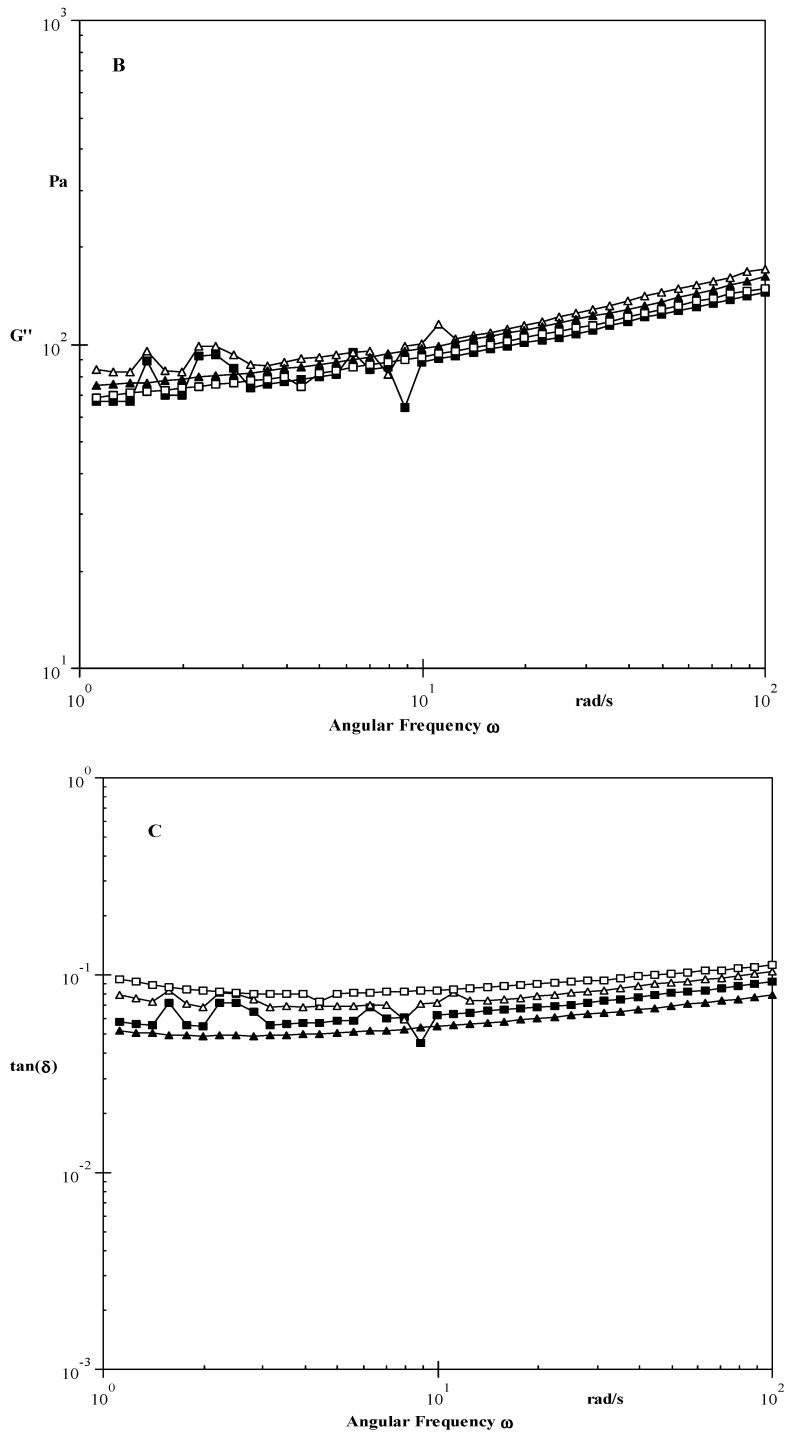
(**A**) Angular frequency dependence of G′ at 25 °C for native and modified starches. (**B**) Angular frequency dependence of G″ at 25 °C for native and modified starches. (**C**) Angular frequency dependence of tanδ at 25 °C for native and modified starches. _▀_—Native starch Δ—HMT □—Microwave ▲—Sonication.

During the frequency sweep test G′, G′′, and tanδ values were evaluated and tabulated in Table 4 and Figure 3A–C. The G′ value for the native starch was observed to be 1370 Pa, which was higher than starch treated with HMT (1338 Pa) and MT (1039 Pa), while the reverse was observed for the starch treated with ST (1730 Pa). Sandhu et al. [26] observed a decrease in the G′ value for HMT pearl millet starches. This may be due to the increased deformability of the starch granule or the lesser elasticity of the continuous phase, and this would be associated with a lesser ability to form an amylose gel in the continuous phase [38]. The G″ value varied from 83 to 94 Pa. An increase in the G″ value was noticed after the modifications. The G′ value was observed to be more than the G″ value, indicating the elastic behaviour of the starches. During the observed frequency range (0.1–100 rad/s), no cross-over was found for G′ and G′′, which showed the stability of starch paste for the evaluated range. The tanδ values of starch pastes was observed to be from 0.05 to 0.08. The lesser tan δ value, the more the stronger the gel. tan δ < 1 showed the elastic behaviour of the starch pastes, while a tanδ value above 1 showed viscous paste behaviour. Similar tanδ results were reported for the modified starches [39,40,41].

### 3.5. XRD Pattern

**Figure 4:**
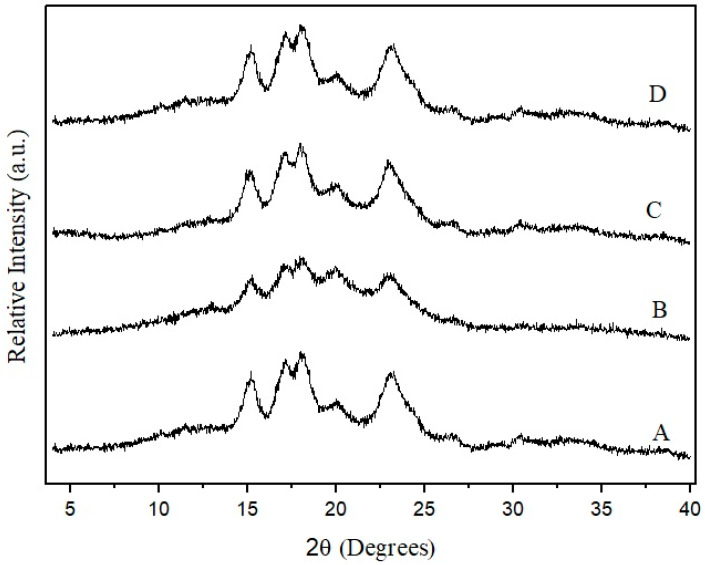
X-ray diffraction patterns of native and modified starches. (**A**) Native starch (**B**) HMT (**C**) Microwave (**D**) Sonication.

The crystalline pattern of the starches was analyzed using XRD, as shown in Figure 4. All starches show an A-type crystalline pattern with peaks at 15°, 17°, 18°, and 23.1 (2θ). This pattern is shown by cereal starches. According to Sandhu and Siroha [42], pearl millet starches showed an A-type crystalline pattern. After modification, no change in the crystalline pattern was observed. Li et al. [15] observed a decrease in relative crystallinity (RC) after MT, and this decrease may be due to changes in the starch structure, including damage to the crystalline regions, disappearance of the double helices, and degradation of the starch granules. ST only affects amorphous regions, and the crystalline region of the starch granule is not sensitive to ST [30,43]. Klein et al. [44] reported a decrease in crystallinity after the HMT. Due to thermal energy, association of adjacent double helices occurs, and number of hydrogen bonds also increases, which results in alterations in the X-ray intensities of cereal starches [6].

### 3.6. Morphological Characteristics

**Figure 5:**
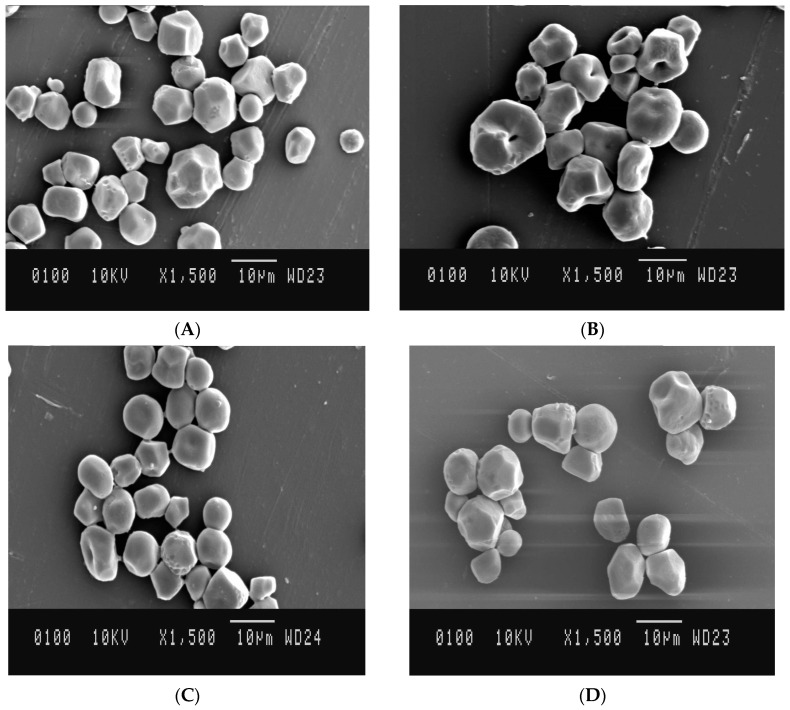
Morphological properties of native and modified starches. (**A**) Native starch (**B**) HMT (**C**) Microwave (**D**) Sonication.

SEM shows the starch granules varying in size from small to large, different shapes (spherical & polygonal), and some starch granules are shown to have some pore like structures on the granule surface (Figure 5). After HMT and MT, significant effects were observed on the surfaces of the starch granules. After HMT treatment, cavities and indentation were observed. Microwave treated starch also showed some cavities on the granule surface, but these changes were less than those of HMT starch. No significant effects were observed for ST starch. Variation in the surface characteristics of starch granules after MT has been reported to depend on the degree of gelatinization that occurs during microwave heating [45]. Liu et al. [46] reported that partial gelatinization of starch occurs due to HMT results in the formation of cavities, fissures, and holes on the surface of starch granules. It can be noticed that MT was able to create cracks on the granules with the simultaneous reduction in moisture content favouring shrinkage of starch granules.

### 3.7. Particle Size Distributions

The particle size distributions of the starches are tabulated in Table 5. In this investigation, d (4,3) shows the mean volume diameter; d (3,2) characterizes the area mean diameter; and d (10), d (50), and d (90) denote the number of starch granules that are smaller (i.e., 10%, 50%, and 90%, respectively) than the average starch granule in the sample. The mean area diameter and mean volume diameter of the starches varied from 8.24 to 17.6µm and 16.6 to 43.1 µm, respectively. After HMT and MT, the mean area and mean volume diameters increased. After HMT and MT, d (10), d (50), and d (90) values increased, which showed that the average granule size increased, while the reverse was observed for starch treated with ST. After physical treatment, the largest increase in the granule size was evaluated for HMT starch. Molavi et al. [47] observed that HMT increased the aggregation of starch granules.

### 3.8. Properties of Starch Films

Properties of native and modified starch films are tabulated in Table 6. The moisture content of films varied from 23.54 to 27.11%. After the modification, the moisture content of films decreased, and the largest decrease was observed for the film prepared from HMT starch. Film thickness is an important factor because of its barrier characteristics as well as its water vapour permeability [48]. Film thickness varied from 0.099 to 0.105 mm. Solubility is one of the most important parameters for food packaging materials, and on the basis of the properties of different foods, there is a need to choose packaging materials with different solubility. Ilyas et al. [49] suggested that packaging for high moisture food required packaging material of low water solubility capacity. Film solubility varied from 34.04 to 37.55%. The largest value was observed for film prepared using microwave treated starch. The opacity ranged from 1.744 to 2.812%. The smallest value was observed for films prepared using native starch. The opacity of the starch films is important when the films are applied as food surface coatings when the packaged food is displayed to the customer.

## 4. Conclusions

In present study, pearl millet starch was modified using HMT, MT, and ST. Significant (*p* < 0.05) variation in the characteristics of the modified starches were observed based on the type of treatment provided to the starch. After modification, SP decreased for starch treated with HMT and MT, while solubility power increased after modification. Modified starches showed higher transition temperature compared to native starch. The pasting and rheological properties were also altered after treatment. The values of PV and G′ decreased after HMT and MT while reverse was observed for starch treated with ST. The morphological properties of the starch treated with ST showed the least effect on the granule surface, while starch granules treated with HMT had the highest effect. Films prepared using modified starches showed less moisture and higher solubility and opacity content compared to films prepared using native starch. The findings suggest that physical modifications can improve the stability and gel texture of starches, which will improve the applications of starches.

## Figures and Tables

**Table 1:** Physicochemical properties of native and modified starches.

Sample	Amylose Content (%)	Swelling Power (g/g)	Solubility (%)
Native	18.2 ± 0.3 ^c^	15.86 ± 0.5 ^b^	25.2 ± 0.2 ^a^
HMT	14.85 ± 0.2 ^b^	12.64 ± 0.4 ^a^	26.4 ± 0.3 ^b^
Microwave	14.32 ± 0.4 ^a^	12.63 ± 0.6 ^a^	28.8 ± 0.2 ^c^
Sonication	19.5 ± 0.3 ^d^	17.53 ± 0.4 ^c^	26.2 ± 0.1 ^b^

Means followed by the similar superscript within the column do not differ significantly (*p* < 0.05).

**Table 2:** Thermal properties of native and modified starches.

Sample	To (°C)	Tp (°C)	Tc (°C)	∆H_gel_ (J/g)	PHI	R
Control	62.92 ± 0.5 ^a^	67.95 ± 0.2 ^a^	73.78 ± 0.2 ^a^	10.8 ± 0.1 ^d^	2.15 ± 0.02 ^b^	10.06 ± 0.3 ^bc^
HMT	76.16 ± 0.3 ^d^	81.05 ± 0.4 ^d^	84.50 ± 0.3 ^d^	7.9 ± 0.3 ^a^	1.75 ± 0.04 ^a^	9.78 ± 0.3 ^b^
MIC	72.15 ± 0.4 ^c^	76.65 ± 0.3 ^c^	81.10 ± 0.3 ^c^	8.6 ± 0.2 ^b^	1.91 ± 0.05 ^ab^	9.0 ± 0.2 ^a^
Sonication	63.40 ± 0.3 ^b^	68.35 ± 0.5 ^b^	74.11 ± 0.5 ^b^	9.2 ± 0.2 ^c^	1.86 ± 0.03 ^a^	9.9 ± 0.5 ^b^

Means followed by the similar superscript within the column do not differ significantly (*p* < 0.05).

**Table 3:** Pasting properties of native and modified starches.

Sample	PV (mPa·s)	BV (mPa·s)	TV (mPa·s)	SV (mPa·s)	FV (mPa·s)	PT (°C)
Native	995 ± 20 ^c^	443 ± 6 ^c^	552 ± 10 ^d^	311 ± 8 ^c^	863 ± 11 ^d^	72.8 ± 0.2 ^a^
HMT	536 ± 12 ^a^	74.0 ± 5 ^a^	462 ± 9 ^c^	274 ± 5 ^b^	736 ± 9 ^b^	75.2 ± 0.1 ^c^
Microwave	630 ± 15 ^b^	209 ± 7 ^b^	421 ± 7 ^a^	266 ± 6 ^a^	687 ± 10 ^a^	77.0 ± 0.2 ^d^
Sonication	1034 ± 11 ^d^	586 ± 8 ^d^	448 ± 9 ^b^	389 ± 8 ^d^	837 ± 12 ^c^	73.4 ± 0.1 ^b^

Means followed by the similar superscript within the column do not differ significantly (*p* < 0.05).

**Table 4:** Rheological properties of native and modified starches during frequency sweep test.

Sample	(G′) (Pa)	G′′ (Pa)	tanδ
Native	1370 ± 15 ^c^	83 ± 5 ^a^	0.06 ^a^
HMT	1338 ± 11 ^b^	94 ± 4 ^bc^	0.07 ^a^
Microwave	1039 ± 12 ^a^	84 ± 6 ^a^	0.08 ^a^
Sonication	1730 ± 16 ^d^	90 ± 5 ^b^	0.05 ^a^

Means followed by the similar superscript within the column do not differ significantly (*p* < 0.05).

**Table 5:** Particle size distribution of native and modified starches.

Sample	D(3,2) (µm)	D(4,3) (µm)	Dv (10) (µm)	Dv (50) (µm)	Dv (90) (µm)
Native	8.43 ± 0.01 ^b^	17.0 ± 0.03 ^b^	6.52 ± 0.01 ^ab^	13.9 ± 0.02 ^b^	29.1 ± 0.03 ^b^
HMT	17.6 ± 0.02 ^d^	43.1 ± 0.05 ^d^	9.90 ± 0.02 ^c^	36.4 ± 0.04 ^d^	86.1 ± 0.05 ^d^
Microwave	9.91 ± 0.02 ^c^	20.0 ± 0.02 ^c^	7.13 ± 0.02 ^b^	16.4 ± 0.02 ^c^	36.4 ± 0.03 ^c^
Sonication	8.24 ± 0.01 ^a^	16.6 ± 0.03 ^a^	6.41 ± 0.03 ^a^	13.1 ± 0.03 ^a^	27.6 ± 0.02 ^a^

Means followed by the similar superscript within the column do not differ significantly (*p* < 0.05).

**Table 6:** Moisture content, thickness, water solubility, and opacity of native and modified starch films.

Sample	Moisture Content (%)	Thickness (mm)	Water Solubility (%)	Opacity (%)
Native	27.11 ± 1.6 ^c^	0.105 ± 0.003	34.04 ± 2.1 ^a^	1.744 ± 00 ^a^
HMT	23.54 ± 2.2 ^a^	0.101 ± 0.002	35.10 ± 1.8 ^b^	2.812 ± 00 ^d^
Microwave	23.80 ± 2.0 ^ab^	0.099 ± 0.001	37.55 ± 2.8 ^c^	2.710 ± 00 ^c^
Sonication	26.92 ± 1.5 ^b^	0.104 ± 0.002	35.40 ± 2.2 ^b^	2.191 ± 00 ^b^

Means followed by the similar superscript within the column do not differ significantly (*p* < 0.05).

## Data Availability

Not applicable.
